# A Critique of the Attentional Window Account of Capture Failures

**DOI:** 10.5334/joc.270

**Published:** 2023-07-06

**Authors:** Nicholas Gaspelin, Howard E. Egeth, Steven J. Luck

**Affiliations:** 1 Department of Psychology, State University of New York at Binghamton, US; 2 Department of Psychological and Brain Sciences, Johns Hopkins University, US; 3 Center for Mind and Brain, University of California, Davis, US

**Keywords:** attention, attentional capture, suppression, attentional window account

## Abstract

There has been a lengthy debate about whether salient stimuli have the power to automatically capture attention, even when entirely task irrelevant. Theeuwes (2022) has suggested that an attentional window account could explain why capture is observed in some studies, but not others. According to this account, when search is difficult, participants narrow their attentional window, and this prevents the salient distractor from generating a saliency signal. In turn, this causes the salient distractor to fail to capture attention. In the present commentary, we describe two major problems with this account. First, the attentional window account proposes that attention must be focused so narrowly that featural information from the salient distractor will be filtered prior to saliency computations. However, many previous studies observing no capture provided evidence that featural processing was sufficiently detailed to guide attention toward the target shape. This indicates that the attentional window was sufficiently broad to allow featural processing. Second, the attentional window account proposes that capture should occur more readily in easy search tasks than difficult search tasks. We review previous studies that violate this basic prediction of the attentional window account. A more parsimonious account of the data is that control over feature processing can be exerted proactively to prevent capture, at least under certain conditions.

Theeuwes (2022) outlines the *attentional window account* as a potential resolution to a long-standing debate about the interaction of top-down and bottom-up processes in shaping the guidance of visual attention. This account claims that salient stimuli will automatically capture attention unless the attentional window is small. More precisely, if attention is broadly distributed at stimulus onset (in preparation for a parallel search), feature information from all objects in the display is processed, allowing the most salient object to capture attention. By contrast, if attention is narrowly focused at stimulus onset (in preparation for a serial search), only the features at the attended location are fed forward to later processing stages and this will prevent the features of the most salient object from being processed and thereby capturing attention. This account is meant to explain a large number of previous studies that have shown that salient distractors can be ignored under conditions that promote feature-based attentional guidance by using heterogenous search displays. Although the attentional window account is an interesting and creative take on past findings, we find that it implies a model of attention that is implausible and inconsistent with previous results. In the current paper, we will describe two major shortcomings of this account.

## Is the Attentional Window Narrowly Focused with Heterogenous Distractors?

The idea that attention can be either broadly or narrowly focused is well supported by previous research (e.g., Eriksen & St. James, 1986; LaBerge, 1983; Leonard et al., 2013), but this mechanism cannot easily explain the findings that Theeuwes (2022) seeks to explain. For the attentional window account to work, a few assumptions must be made. First, it is necessary to assume that attention is narrowly focused prior to the onset of the stimulus array. Otherwise, the features of the salient distractor would be available to preattentive processing and saliency computations, leading to attentional capture. Second, it would be necessary to assume that the stimulus array—a large and massively salient stimulus—does not automatically broaden the focus of attention (Castiello & Umiltà, 1990). Given that attentional allocation is strongly determined by competition (Desimone & Duncan, 1995), it would be challenging for observers to maintain a narrow focus of attention immediately following the onset of a large search array even if they were highly motivated to do so.

Crucially, if a narrow attentional focus prevents the processing of feature information at unattended locations, then it would not be possible for featural information to guide attention. In other words, if there is enough featural information present to guide attention to items with task-relevant features, then there is certainly enough featural information present to determine that one of the distractors is a highly salient color singleton. Thus, for the attentional window account to be feasible, attention must be focused so narrowly that the first shift of attention after stimulus onset is completely random. Although it may be possible to design a search task that is so difficult that the first shift of attention is completely random, most of the tasks classified as “serial” by Theeuwes (2022) contain enough clear featural information to allow observers to perform a guided search. Given the massive evidence for feature-based guidance of attention (Wolfe & Horowitz, 2004), it seems unlikely that observers would make a random shift of attention when featural information is present that could guide that first shift. This creates what we call the *guidance problem*: if enough featural information is present to guide attention to the target stimulus, then there should be enough featural information for saliency to be computed and control attention (unless the salient object was being suppressed, as proposed by the signal suppression hypothesis; for a review, see Gaspelin & Luck, 2018c).

**Figure 1:**
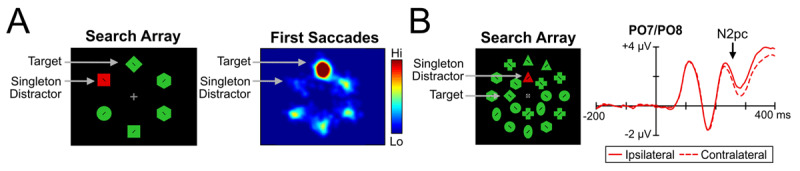
Previous studies showing guidance toward the target shape, which seems indicates that featural information was not filtered by focusing attention as claimed by the attentional window account. **(A)** First eye movements from Gaspelin et al. (2017, Exp. 3) clearly showed a bias toward the target shape above levels of the other nonsingleton distractors. Specifically, the heat map shows that the vast majority of first saccades went to the target location. **(B)** ERPs from Stillwell et al. (2022, Exp. 2) show clear evidence of an N2pc elicited by the target shape, suggesting that the target shape was preferentially attended compared to other shapes.

In many previous studies in which Theeuwes (2022) would argue that the attentional window was too narrow to allow capture, attention *was* preferentially guided toward search items with target features. This suggests that featural information was available to the attentional system and was not shielded as claimed by the attentional window account. For example, Gaspelin et al. (2017, Exp. 3) measured eye movements in an *additional singleton paradigm* in which participants searched for a specific shape and were instructed to ignore color singleton distractors (Figure 1A). The first saccade was directed to the target shape on over 45% of trials and was directed to any given nonsingleton distractor on 12% of trials. However, the salient singleton did not capture attention and was actually suppressed: It was fixated on only 5% of trials when present, and the presence of the singleton did not slow the allocation of attention to the target (see also Adams et al., 2022; Gaspelin et al., 2019; Gaspelin & Luck, 2018a; Hamblin-Frohman et al., 2022). It does not seem plausible that enough featural processing occurred to guide attention toward the target shape, and yet there was not enough featural processing for the saliency of the color singleton to be calculated.

Other approaches have also demonstrated similar evidence of attentional guidance toward the target shape in search tasks with heterogenous distractors. For example, Gaspelin et al. (2015, Exp. 2) had participants search for a target shape amongst heterogenous shapes. On a portion of trials, probe letters were briefly superimposed on search items and participants attempted to recall as many letters as possible. Probe recall was higher for the target shape (57%) than the baseline level of the nonsingleton distractor shapes (15%). This clearly suggests that the initial shift of attention was guided toward objects with the target features, and yet capture by a salient distractor was not observed (see also Gaspelin & Luck, 2018a; Lien et al., 2022; Stilwell & Gaspelin, 2021). Similarly, several ERP studies using heterogenous displays show a rapid N2pc to the target stimulus, suggesting feature-based guidance, but with no evidence of capture by a salient distractor (see Figure 1B from Stilwell et al., 2022; see also Gaspelin & Luck, 2018b; Barras & Kerzel, 2016). These findings challenge the attentional window account because they indicate that there was enough featural processing to allow the target shape to be rapidly attended, and yet the salient color singleton did not capture attention.

## Does the Attentional Window Account Predict Which Tasks Yield Capture?

Even if we ignore the evidence that the attentional window was broad enough for substantial feature processing in previous experiments, the attentional window account does a poor job of accounting for the presence or absence of capture in prior studies. A key tenet of the attentional window account is that easy search tasks that encourage a “parallel” search will yield attentional capture, and difficult search tasks that encourage a “serial” search will not yield capture. A natural question is therefore whether this prediction fits the general pattern of results observed in the existing attentional capture literature.

For example, a distinction between easy and difficult search tasks cannot account for the lack of capture by salient-but-irrelevant cues in the spatial-cueing paradigm (e.g., Folk et al., 1992; Folk & Remington, 1998). In this task, participants search for a salient target stimulus (e.g., red letter) amongst distractors that are dissimilar to the target (e.g., green and white letters). Before the search array appears, a salient precue appears at a randomly selected location. If this cue captures attention, it should produce a cue validity effect, whereby RTs are faster on trials where the precue appears at the target location (valid trial) than when precue appears at a nontarget location (invalid trial). The canonical finding is that salient cues do not produce cue validity effects unless they match the features of the target (Folk et al., 1992; Folk & Remington, 1998, 2006; Lien et al., 2008; Lien, Ruthruff, & Cornett, 2010; Lien, Ruthruff, & Johnston, 2010).

The lack of capture in the spatial-cueing paradigm is problematic for the attentional window account because these tasks typically use easy search displays that would seem to encourage a diffuse attentional window. For example, Lien et al. (2010) measured search slopes in a prototypical spatial-cueing paradigm and found search slopes of 6 ms/item, which would seem to indicate an easy “parallel” search. Thus, there is no reason to expect that the attentional window is focused in preparation for a difficult search at the time of the cue display in a manner that prevents capture by a target-mismatching cue but not a target-matching cue. The lack of capture by salient cues also cannot be attributed to rapid disengagement of attention from the salient cue before the search array appears (Folk & Remington, 2010).^1^ Furthermore, many spatial-cueing tasks have shown that, if anything, cue validity effects increase with search difficulty, contrary to the predictions of the attentional window account (e.g., Gaspelin et al., 2016; Lamy et al., 2018; Ruthruff et al., 2020).

An easy-difficult search dichotomy also does not explain strategic changes in performance in the additional singleton paradigm. Leber and Egeth (2006b) directly tested the attentional window account. They took advantage of the fact that once an attentional set is established, it tends to persist (Leber & Egeth, 2006a). They trained some participants on displays that varied the target shape from trial to trial, in a homogeneous background. This forced participants to look for shape singletons (singleton-detection mode). Other participants were trained on displays that combined a constant target shape with heterogeneous distractors, which discouraged a strategy of looking for singletons (feature-search mode). In the test phase, all participants were treated identically. They searched displays where the target was salient, but either search strategy was possible (i.e., the target shape was fixed, allowing feature-search mode, but it was a singleton in the shape dimension, allowing singleton-detection mode). Capture effects during the test phase were greater for participants trained on singleton-detection mode (a 20-ms cost) than participants trained on feature-search mode (a nonsignificant 6-ms cost). Importantly, the search task was extremely easy in both groups, as evidenced by flat search slopes (<2 ms/item), indicating a broad attentional window. This indicates that participants in the feature condition were able to avoid being distracted by a salient stimulus while maintaining a broad attentional window, thus undermining the attentional window account.

A parallel-serial dichotomy also cannot explain the discrepant findings between bottom-up theories of capture and the signal suppression hypothesis (as suggested by Theeuwes, 2022). To briefly recap, Gaspelin et al. (2015) originally used a capture-probe paradigm to demonstrate that salient color singletons could be suppressed below baseline levels. Wang and Theeuwes (2020) later demonstrated that increasing the set size of the displays from 6 to 10 items (to boost the salience of the singleton) caused the singleton to produce (slight) evidence of capture. Stilwell and Gaspelin (2021) then found that this result was due to a design issue that caused floor effects in the probe technique of Wang and Theeuwes. When this problem was eliminated, the singleton distractors were suppressed rather than capturing attention.

**Figure 2:**
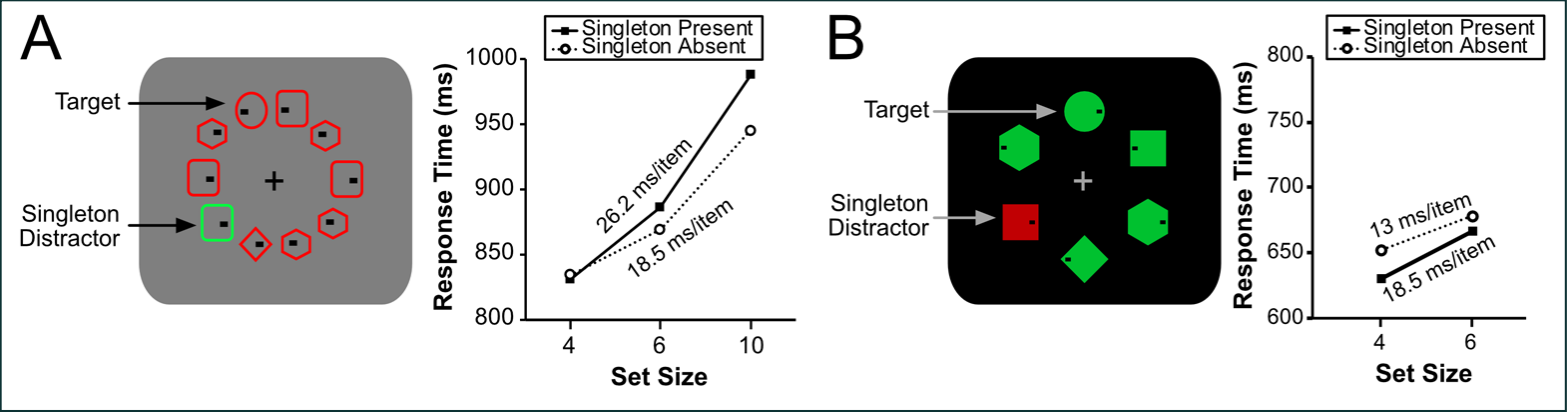
Search displays and search slopes for **(A)** Wang and Theeuwes (2020) and **(B)** Gaspelin et al (2015). Both searches produced steep slopes indicative of a difficult search. Yet, these studies obtained differing results regarding attentional capture by a salient distractor.

Theeuwes (2022) now suggests that the differing results between those of Gaspelin and colleagues and those of Wang and Theeuwes (2020) are due to differences in search strategy. There are two reasons to doubt this claim. First, both sets of studies used a nonsalient target shape (i.e., circle/diamond) that appeared amongst heterogenous distractor shapes, so there is no theoretical reason to believe that the two studies led to different search strategies. Second, as shown in Figure 2, both Wang and Theeuwes (2020) and Gaspelin et al. (2015) had relatively steep search slopes (18.5–26.2 ms/item, and 13.0–18.5 ms/item, respectively). If anything, the search slopes were shallower in Gaspelin et al. (2015). Furthermore, Stilwell and Gaspelin (2021, Exp. 4) used *exactly the same stimuli* as Wang and Theeuwes (2020) and still found evidence of suppression when the floor effect was eliminated. Thus, there is little reason to suspect that an easy-difficult search dichotomy could explain the discrepant results of these studies.

An easy-difficult dichotomy also does not easily explain learned suppression effects, whereby individuals learn to avoid capture by salient distractors on the basis of their specific feature values (Vatterott & Vecera, 2012; see also Gaspelin & Luck, 2018a; Gaspelin et al., 2019; Stilwell et al., 2019; Ramgir & Lamy, in press). For example, Vatterott and Vecera (2012) used displays that are similar to those shown in Figure 1A, in which a target shape (e.g., diamond) appeared amongst heterogenous shapes and the target color remained constant for the entire experiment. The color of the singleton distractor was constant within a block but changed across blocks (e.g., from red to yellow). In the first half of each block, the singleton captured attention. In the second half of each block, capture was eliminated. This pattern was interpreted to suggest that individuals learned to suppress the upcoming singleton based upon its specific color. This result is not easily explained by the attentional window account because (a) capture occurred in the first half of each block under a seemingly difficult search, and (b) capture disappeared as participants gained experience with the specific color of singleton distractor despite no apparent change in search difficulty. In other words, there is no reason to suspect that learning the singleton’s color value would cause a narrowing of the attentional window.

## Conclusion

In sum, the attentional window account is unrealistic for two main reasons. First, it presumes that the attentional window is so narrowly focused at search display onset that feature processing is suppressed outside the window. However, this seems unlikely on theoretical grounds, and it is also inconsistent with clear evidence of feature-based guidance. Second, it presumes that the degree of capture should vary across studies with the difficulty of the search task. However, this does not fit the pattern observed in prior research. In our view, a more parsimonious model of attention would simply accept that salient distractors can sometimes be prevented by feature-based attentional control settings (e.g., Folk et al., 1992; Bacon & Egeth, 1994; Gaspelin et al., 2015).

## Footnotes

An argument advocated by Theeuwes and colleagues is that the results of the spatial-cueing paradigm can be ignored because capture is immediately followed by rapid disengagement from the salient cue (Theeuwes et al., 2000). But there is now strong evidence against this viewpoint (for a review, see Folk & Remington, 2010; see also Chen & Mordkoff, 2007; Folk & Remington, 2006).
